# Inter-rater agreement between radiologists using the novel CT-TDV (T3c+; tumour deposits; EMVI) system in patients with potentially curable right colon cancer

**DOI:** 10.1259/bjr.20220682

**Published:** 2023-04-12

**Authors:** Nicola Hodges, Oliver Duxbury, Alison Corr, Seung Hyon Cho, Danilo Miskovic, Gina Brown

**Affiliations:** 1 St Mark’s Hospital, Harrow, London, United Kingdom; 2 Imperial College London Hammersmith Campus, London, United Kingdom; 3 Basingstoke and North Hampshire Hospital, Basingstoke, United Kingdom; 4 Kyungpook National University, Daehak-ro, Buk-gu. Daegu, South Korea

## Abstract

**Objectives:**

The novel CT-TDV scoring system, identifying T3*c* + disease; the presence/absence of tumour deposits and EMVI has been shown to be superior in predicting prognosis when compared to the CT-TNM staging system in the evaluation of colon cancer. Reproducibility of this scoring system between specialist GI radiologists has not been assessed previously. The aim of this study was to assess the inter-rater agreement of gastrointestinal radiologists in assessing the novel pre-operative CT-TDV scoring of patients with potentially curable right-sided colon cancer.

**Methods:**

Ninety-three right colon cancer pre-operative CT scans were graded as CT TDV “good” versus TDV “poor” by four radiologists. Inter-rater agreement was assessed using the intraclass correlation coefficient (ICC) between all four readers and individual readers against the central radiologist using Cohen’s κ statistic.

**Results:**

The ICC comparing those graded as TDV “good” versus TDV “poor” for all 93 cases was 0.61 (0.51–0.70) indicating moderate reliability. Individual κ scores across the 93 cases were 0.76, 0.59 and 0.59 (*p* < 0.001) indicating moderate to substantial agreement.

**Conclusion:**

The CT TDV scoring system is reproducible amongst trained gastrointestinal radiologists in the assessment of newly diagnosed right colon cancer.

**Advances in knowledge:**

This further validates the clinical utility of the CT TDV scoring system as a prognostic tool to guide the management of patients with potentially curable right colon cancer.

## Introduction

Pre-operative radiological staging, in the form of contrast-enhanced chest, abdomen and pelvis Computed Tomography (CT) has, until recently, only been used to determine the presence or absence of distant metastatic disease and local resectability of the primary tumour. With increasing interest and success in the use of neoadjuvant therapy in colon cancer, (FOXTROT, PRODIGE 22),^1–3^ there is an increasing reliance on pre-operative imaging to be able to accurately identify patients with high-risk disease who may benefit from more aggressive treatment while sparing those with low-risk disease the associated morbidity.

To date, pre-operative CT staging of colon cancer has attempted to stratify patients into high- and low-risk groups by trying to predict the pathological TNM stage. Two major problems with this approach are the poor correlation between radiological T/N stage and pathological T/N stage^2–5^ and secondly, the imperfect nature of the pathological TNM staging system at predicting prognosis with significant overlap in survival outcomes between those with pathologically determined stage II and III disease.^6^ Recent studies suggest that this is likely due to an overweighting of the “N” category in the current classification system, while it is also recognised that pathological assessment of important prognostic markers such as extramural venous invasion (EMVI) and tumour deposits (TD) can be insensitive due to limitations in the pathology assessment process with cut-up limitations and the fact it is unrealistic to examine the entire resected colonic mesentery under the microscope.^7^

Radiological staging of colon cancer must therefore be re-evaluated by returning to the basic principles of what pre-operative staging is trying to achieve. Firstly, a valid radiological staging system should be able to predict prognosis, thus identifying a high-risk group of patients who may benefit from more intensive treatment in the form of neoadjuvant therapy or more radical surgery. Secondly, the proposed radiological staging system must be reproducible, that is specialist GI radiologists should be able to agree on which stage is assigned to each patient.

This is of particular importance for patients with potentially curable right-sided colon cancer, whom we know have a worse prognosis compared to equivalent tumours on the left-side-and in whom more extensive surgery in the form of complete mesocolic excision may be beneficial.^8,9^

The CT TDV scoring system, first described by D’Souza et al, may provide this optimal staging system.^10^ In the evaluation of sigmoid cancers, they were able to demonstrate improved prognostic performance using a CT scoring system identifying T3*c* + disease, the presence or absence of EMVI and the presence or absence of tumour deposits over the classical ctTNM staging. To our knowledge, the reliability of this system has not been assessed with evaluation of inter-rater agreement between radiologists.

The aim of this study was to assess the inter-rater agreement of GI radiologists in assessing the novel pre-operative CT TDV score of patients with potentially curable right-sided colon cancer.

## Methods

### Patient population, Radiologists and Training

Three GI radiologists from different centres in the UK and South Korea, with between one to ten years of consultant experience individually, were invited to participate in the study in addition to the central radiologist who first described the scoring system (20 years of consultant experience). A 2-h interactive video-conference teaching session was arranged where the central radiologist explained the principles of the CT-TDV staging system, illustrated by case examples. All four radiologists were then asked to grade a training set of 41 historical anonymised right colon CT scans uploaded to a web-based radiology platform, CIMAR, according to the CT-TDV system. All radiologists were blinded to both the original CT clinical stage and the pathological stage.

After completion of the scoring of the training set of right colon CT scans, a further 2-h interactive video-conference teaching session was held, and cases where there had been disagreement between the radiologists were reviewed. Following this, a further 52 right-colon CT scans were then scored as a “test” set. The “test” set was identified from consecutive patients discussed at respective colorectal MDTs from two NHS Trusts and diagnosed with right-sided colon cancer (caecum, ascending colon, hepatic flexure or transverse colon). Consecutive patients discussed at these MDTs were intentionally chosen so as to represent the typical stage presentations of newly diagnosed, potentially curable right colon cancer and allow generalisability of our study results. Patients were excluded if they had evidence of metastatic disease at the time of staging. Patients who had a staging CT colonography (as opposed to standard CT abdomen/pelvis) were also excluded.

### Scan protocol

Multidetector CT scan images were obtained from a variety of machine manufacturers and models as standardly used in each NHS trust. Portal venous images were used to score the TDV system with a minimum slice thickness of 3 mm and multiplanar reconstruction available in axial, coronal and sagittal sections.

### TDV scoring system

Based on the individual components of the TDV score, each patient was then defined as being TDV “good” or TDV “poor” as described in Table 1.

**Table 1.** Definition TDV “good” *vs* TDV “poor”

TDV good	TDV poor
**T** stage	**T** stage
T1/2	T3c/d
T3a/b	T4a/b
Tumour **D**eposits	Tumour **D**eposits
Absent	Present
EM**V**I	EM**V**I
Absent	Present
And no TDV poor criteria	≥1 criteria = TDV poor

### Definitions

**Figure 1.**
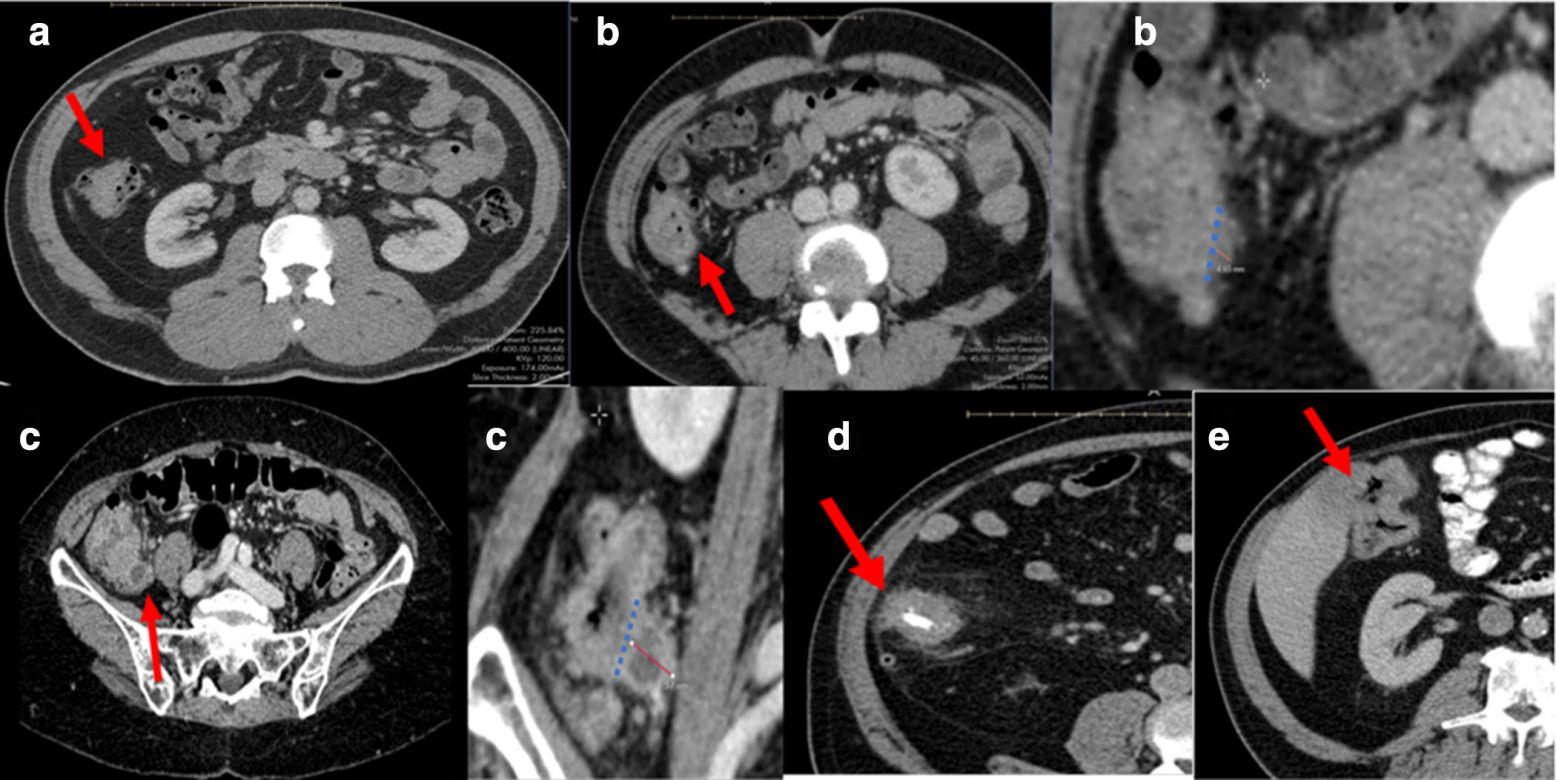
T-stage; A = example of T1/T2 tumour; B = Example of T3a/3b tumour; C= example of T3c/3d tumour; D = example of T4a tumour; E= example of T4b tumour.

**Figure 2.**
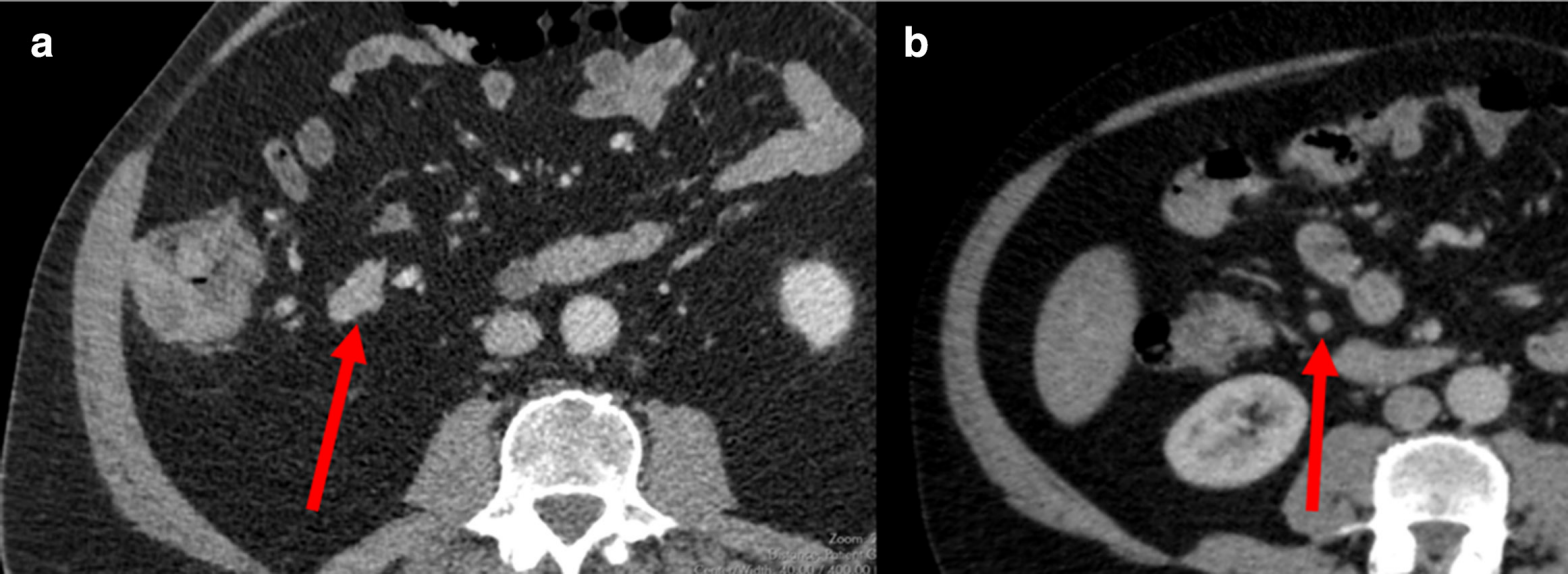
D-status; A = example of a tumour deposit – on serial axial views a vein can be seen entering and leaving the lesion; B = example of lymph node – on serial axial views no vein identified as draining directly into the lesion.

**Figure 3.**
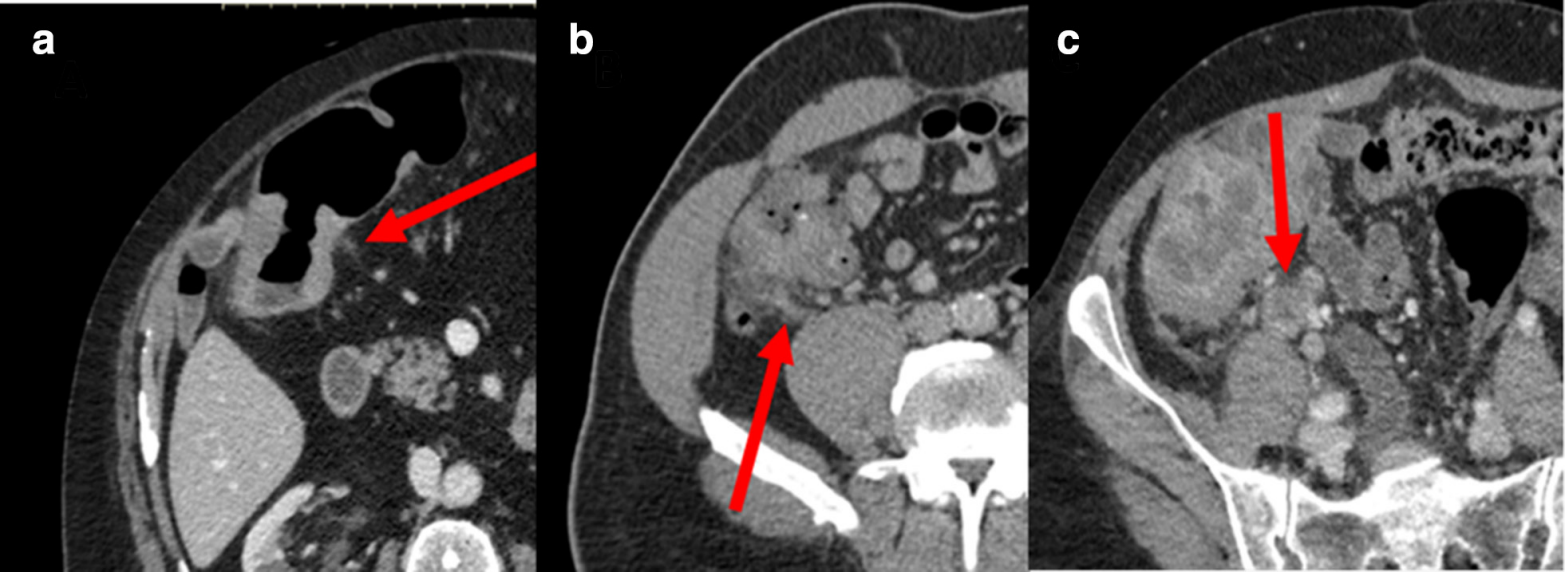
V status; A = Example of irregular small veins/stranding around tumour site (EMVI negative); B = Serpiginous extension of tumour within a peri-tumoural vein (EMVI positive); C = Extensive tumour thrombus plugging vein draining tumour (EMVI positive).

A pictorial guide to the TDV scoring system is shown in Figures 1–3.

### T-stage

Assessment of depth of invasion of tumour. The T-stage was classified as T1/2 if the tumour was assessed as not extending beyond the contour of the muscle coat (muscularis propria); T3a/b if the tumour invaded through the muscularis propria into the subserosa or non-peritonealised pericolic tissue≤5 mm.^11^ To measure extramural depth (EMD), the deepest area of tumour invasion was identified. This image was magnified and EMD measured from the outer border of the muscularis propria to the deepest area of invasion.^12^ T3c/d tumour invades through muscularis propria into subserosa or non-peritonealised pericolic tissue>5 mm. i.e. extramural depth of invasion>5 mm. T4a tumour perforates visceral peritoneum. T4b tumour directly invades other organs or structures.

### D-status

Assessment of presence/absence of tumour deposits. Similar to the appearance of tumour deposits on rectal MRI, tumour deposits were defined as nodules of tumour within the mesocolon, that are discontinuous from the primary tumour mass, and that appear to directly interrupt the course of a vein.^13^ When viewed on CT, tumour deposits will be identified as having a vein draining into and out of the lesion on serial axial, coronal or sagittal images. Tumour deposits often have an irregular or spiculated border.

A lymph node in contrast may be seen adjacent to veins but when seen on two orthogonal views will not interrupt their course.^13^ A lymph node is more likely to have a smooth contour on CT.

For detection of tumour deposits on CT the first step is to identify the lesion in the mesentery. The second step is to interrogate the lesion – does it expand the vessel in two planes? If yes, it is a tumour deposit. If this expansion is within a major draining vein, then this would also be classified as EMVI positive in addition to tumour deposit positive.

### V- status

Assessment of EMVI. EMVI is best assessed on portal venous MDCT. Only macroscopic EMVI can be detected on CT.

### EMVI negative tumours

Characterised by absent peri-tumoural veins or normal calibre veins draining the region of the tumour. Stranding in the vicinity of the tumour consistent with an inflammatory response or desmoplastic reaction and without any of the features subsequently described should be considered EMVI negative.

### EMVI positive tumours

Must have at least one of the following characteristics present: 1) serpiginous extension of the primary tumour consistent with extension of the tumour within a vascular structure, which has resulted in irregular distortion of the vein; 2) expansion of a peri-tumoural vein *i.e.,* a sudden change in calibre of the vein (may be focal or persistent). Often identified as an irregularity/nodularity of the vessel wall. This is as a result of tumour thrombus within the vein. The tumour thrombus itself may not be visualised on CT and therefore only the contour of the vessel wall can be assessed; 3) obvious tumour thrombus/tumour plugging veins draining the tumour.

Significant tumour thrombus within a vein may give the appearance of a tumour deposit. This is due to the underlying pathology with extensive EMVI leading to tumour thrombus and perforation of the tumour thrombus through the vessel wall resulting in the formation of a tumour deposit. In the most advanced cases the lesion would be called both a TD and EMVI positive. Whether the lesion is both a TD and EMVI depends on the calibre of the vein. A TD associated with only small calibre veins would most correctly be called a solitary TD, while those TDs associated with thrombus in a major draining vein would be called both TD positive and EMVI positive. For the purposes of the CT TDV scoring system the nuance of whether the lesion is called EMVI or a tumour deposit is not important as long as the lesion itself is identified.

### Statistical analysis

Inter-rater agreement was assessed by the intraclass correlation coefficient (ICC) between all four readers with ICC<0.5 indicating poor reliability, 0.5–0.75 moderate reliability, 0.75–0.90 good reliability and >0.90 excellent reliability. ICC and their 95% confidence intervals were calculated using IBM SPSS Statistics for Windows, version 28.0. Armonk, NY: IBM Corp based on a single-rater, absolute-agreement, two-way random effects model. The inter-rater reliability of individual readers was also assessed against the central radiologist (assessment previously validated against prognosis) using Cohen’s κ statistic with ≤0 indicating no agreement, 0.01–0.20 slight agreement, 0.21–0.40 fair agreement, 0.41–0.60 moderate agreement, 0.61–0.80 substantial agreement and 0.81–1.00 almost perfect agreement.

Ethics approval for this study was obtained with REC 20/NI/0179. IRAS 287131.

## Results

In total, all 4 radiologists scored 93 right colon cancer CT scans, 41 in the “training set” and 52 in the “test set” (Tables 2–4). The ICC comparing those graded as TDV “good” versus TDV “poor” for all 93 cases was 0.61 (0.51–0.70) indicating moderate reliability. Individual κ scores with the central radiologist across the 93 cases for grading of TDV “good” versus TDV “poor” were 0.76 (88% agreement), 0.59 (80% agreement) and 0.59 (81% agreement)(*p* < 0.001) indicating moderate to substantial agreement (Table 2).

**Table 2.** TDV assessment all cases (*n* = 93)

	Central Radiologist TDV good	Central Radiologist TDV poor
**Rater 1**		
TDV good	37	9
TDV poor	2	45
**Rater 2**		
TDV good	33	13
TDV poor	6	41
**Rater 3**		
TDV good	27	6
TDV poor	12	48

**Table 3.** TDV assessment training cases (*n* = 41)

	Central Radiologist TDV good	Central Radiologist TDV poor
**Rater 1**		
TDV good	18	5
TDV poor	1	17
**Rater 2**		
TDV good	17	6
TDV poor	2	16
**Rater 3**		
TDV good	14	2
TDV poor	5	20

**Table 4.** TDV assessment test cases (*n* = 52)

	Central Radiologist TDV good	Central Radiologist TDV poor
**Rater 1**		
TDV good	19	4
TDV poor	1	28
**Rater 2**		
TDV good	16	7
TDV poor	4	25
**Rater 3**		
TDV good	13	4
TDV poor	7	28

When individual T-stage, D-status and V-status were compared for agreement of assessment of “good” versus “poor” the T-stage ICC was 0.52 (0.41–0.63) with kappas of 0.61 (81% agreement), 0.53 (76% agreement) and 0.66 (83% agreement) (*p* < 0.01); D-status ICC 0.46 (0.36–0.57) with kappas of 0.55 (77% agreement), 0.44 (72% agreement), 0.53 (76% agreement) (*p* < 0.01) and V-stage ICC 0.44 (0.33–0.55) with kappas of 0.47 (75% agreement), 0.44 (73% agreement) and 0.58 (78% agreement) (*p* < 0.01).

Comparison of radiological assessment versus pathology was not the aim of this study as outlined in the discussion. Review of cases with available histology did however confirm a range of clinical stages were assessed as part of this study (pT1/T2 10%, pT3 61.1%, pT4 28.9%; pN0 52.5%, pN1/N2 47.5%; EMVI positive 35.6%).

## Discussion

We have demonstrated, for the first time,moderate inter-rater reliability in the ability of GI radiologists to score right colon cancer using the CT-TDV technique.

As this scoring system is novel, to determine if this level of agreement is satisfactory to advocate more widespread adoption of this technique, we must compare our results to studies that have assessed the inter-rater agreement between radiologists of the CT TNM staging system in colon cancer, the current standard used to radiologically stage colon cancer.

The vast majority of studies have assessed inter-rater agreement of the ctTNM staging system using Cohen’s κ between two radiologists and have focused on agreement of the individual components of the staging system as opposed to overall agreement of high versus low-risk groups.^12,14–17^ This is somewhat understandable as in most applications of the ctTNM staging system it is the ‘N” stage (negative *vs* positive) which defines a patient as being low or high risk. Wide variability in agreement is reported in the literature with T-stage kappas between 0.214 and 0.836, N-stage kappas 0.314–0.806 and EMVI kappas 0.178–0.239.^11,12,14,18^. Our results therefore compare favourably for both the individual assessment of the TDV staging system as well as the overall differentiation into TDV “good” and TDV “poor” prognostic groups which would ultimately guide patient management should this approach be adopted more widely in the future.

One of the main benefits of the CT TDV system is that it removes the “ctN” staging from prognostic stratification. It is now widely accepted that CT prediction of nodal involvement (whatever the methodology) in the staging of colon cancer is inaccurate and indeed in previous studies has been likened to having an accuracy of flipping a coin.^12,19^ Furthermore, the weighting of pathological nodal involvement in the TNM staging of colon cancer is likely responsible for the overlap in survival outcomes (survival paradox) between patients with pathological stage II and III disease which does not adequately take into account the poor prognostic attributes EMVI and tumour deposits.^6^ Leijssen et al showed that EMVI positive stage II patients had worse survival outcomes compared with stage III EMVI negative patients, even after adjusting for the use of adjuvant chemotherapy, a finding that has been replicated.^20^ We agree with other authors who have concluded that CT N staging is an inappropriate tool to select patients with colon cancer appropriate for neoadjuvant therapy.^19^ The CT appearance of tumour deposits in colon cancer has not previously been defined. It is likely that previous studies have included tumour deposits in their radiological assessment of “N” stage further complicating the comparison of our study with previous work.

Remarkably only three studies have directly compared radiological CT staging with patient prognosis and outcomes.^10,11,21^ The vast majority have used the accuracy of radiological staging in predicting pathological staging as a surrogate for patient outcomes.

With 93 CT scans assessed by four radiologists, this is one of the largest series assessing inter-rater agreement in the staging of colon cancer and the first to assess inter-rater agreement of the CT TDV system. By including consecutive patients discussed at colorectal multidisciplinary team meetings across 2 NHS trusts, incorporating both district general hospital as well as tertiary referrals, with an even distribution of good and poor prognosis cases,these results are likely generalisable to GI radiologists in multidisciplinary teams (MDTs) throughout the UK and internationally.

A potential limitation to our results is that only GI radiologists scored the CT scans. This may mean our results are not generalisable to radiologists without such subspecialty training. It is unlikely, however, that this has significant clinical implications as it is GI radiologists who re-review CT images in MDTs prior to any decisions being made regarding patient management. The three radiologists, excluding the central radiologist, had no experience reporting the CT TDV system prior to this study. We have demonstrated that adequate agreement can be achieved with minimal further training. This study did not compare radiological assessment with pathological assessment. This was intentional due to the limitations already discussed.

In conclusion,using identical criteria that has previously been proven to be prognostically superior to the ctTNM staging system in sigmoid colon cancer,^10^ we have now shown that the CT TDV scoring system is highly reproducible amongst trained radiologists in the assessment of patients with right colon cancer.
